# Miniaturization does not change conserved spider anatomy, a case study on spider *Rayforstia* (Araneae: Anapidae)

**DOI:** 10.1038/s41598-023-44230-3

**Published:** 2023-10-11

**Authors:** E. A. Propistsova, A. A. Makarova, K. Y. Eskov, A. A. Polilov

**Affiliations:** 1 Faculty of Biology, Lomonosov Moscow State University, Moscow, Russia; 2 Borissiak Paleontological Institute of the Russian Academy of Sciences (PIN), Moscow, Russia

**Keywords:** Entomology, Evolution

## Abstract

Miniaturization is an evolutionary trend observed in many animals. Some arachnid groups, such as spiders and mites, demonstrate a strong tendency toward miniaturization. Some of the most miniaturized spiders belong to the family Anapidae. In this study, using light and confocal microscopy and 3D modelling, we provide the first detailed description of the anatomy of a spider of the genus *Rayforstia*, which is only 900 µm long. In comparison with larger spiders, *Rayforstia* has no branching of the midgut in the prosoma and an increased relative brain volume. In contrast to many miniature insects and mites, the spider shows no reduction of whole organ systems, no allometry of the digestive and reproductive systems, and also no reduction of the set of muscles. Thus, miniature spider shows a more conserved anatomy than insects of a similar size. These findings expand our knowledge of miniaturization in terrestrial arthropods.

## Introduction

Spiders are a very diverse group, comprising over 51 000 known species^1^. They occupy different niches and include orb- (e.g. Araneidae) and sheet-weavers (e.g. Linyphiidae), active predators (e.g. Salticidae), ambush predators (e.g., Thomisidae), and even phytophagous (*Bagheera kiplingi* G. W. Peckham & E. G. Peckham, 1896). Their size varies over a broad range, from the largest tarantula and huntsman spiders (Theraphosidae and Sparassidae) with a limb span of 30 cm^2^ to the smallest Symphytognathidae^3,4^, measuring less than 1 mm^5^. Despite the diverse ecology and size variation of spiders, their anatomy is relatively conserved^6^.

The tagmosis of spiders remains almost unchanged in both the ancestral Mesothelae and in the descendant Opisthothelae, in contrast to mites, which have many tagmosis variants^7^. The organ set of spiders is fairly stable^8,9^, with a few exceptions such as the respiratory system^10–12^ and the spinning organs. Also, there are minor ecology-related variations, such as the change in cheliceral musculature in Palpimanoidea^13^, and changes in venom glands in Scytodidae^14^.

Miniaturization affects the anatomy of all organ systems in terrestrial invertebrates^15^. The apparent limits to miniaturization vary between taxa^16^. Although they are as small as some unicellular organisms, miniature arthropods retain complex anatomy and even complex behaviours^17,18^.

The whole body anatomy of the smallest species has recently been studied in detail in insects^15,19,20^, springtails^21^, and mites^22^, but not in spiders, which are one of the most speciose order of arachnids. Spiders and mites show much variation in size, and the smallest spiders exhibit evolutionary trends parallel to those of mites, such as the reduction or loss of receptors or simplification of the circulatory systems^5^.

The smallest spiders belong to the family Symphytognathidae, which includes the smallest known spider *Patu digua* Forster & Platnick, 1977, measuring 370 µm^3^. Another species of the same genus, *Patu marplesi* Forster, 1959, is 400 µm long. The known material for these species is represented only by the type specimens, making it impossible to study their anatomy. In addition to symphytognathids, there are miniature spiders in the families Anapidae, Oonopidae, Linyphiidae and Theridiidae, but their size is about 1 mm. The family Anapidae (including the subfamily Micropholcommatinae, formerly a separate family) includes 58 genera and 614 species, mostly under 2 mm in size^1^. Anapidae have a worldwide distribution, with most species occurring in South America, Africa, Asia, Australia and New Zealand. Several genera occur in North America and Europe. Spiders live in moist environments, among mosses and leaf litter, sometimes weaving small (3 cm in diameter) webs^23,24^.

There are no studies describing all organ systems of tiny spiders, although some organ systems have been described in separate publications. Juvenile instars of minute spiders show a decreased diameter of neuronal bodies, the CNS entering the coxae of the legs, and the sternum of the thorax convex due to an increase in CNS volume^25,26^. The respiratory system of the smallest anapids^10,12,23^ might be represented by the anterior and posterior spiracles, only by the anterior ones and sometimes with reduced book lungs. The reproductive system of Anapidae is similar to that of other entelegyne spiders and has been described in taxonomic studies, particularly by Rix and Harvey^27^.

Given the relative conservative spider anatomy, we hypothesise that miniaturization may not have such a major impact on it. In this study, we (a) analyze the anatomy of the anapid spider *Rayforstia* cf. *raveni* Rix & Harvey, 2010 using methods of histology, light and confocal microscopy, and 3D reconstruction, (b) compare the anatomy of *Rayforstia* with the anatomy of larger spiders, and (c) compare the miniaturization-related features characteristic of spiders, mites and insects.

## Results

### General anatomy

**Figure 1:**
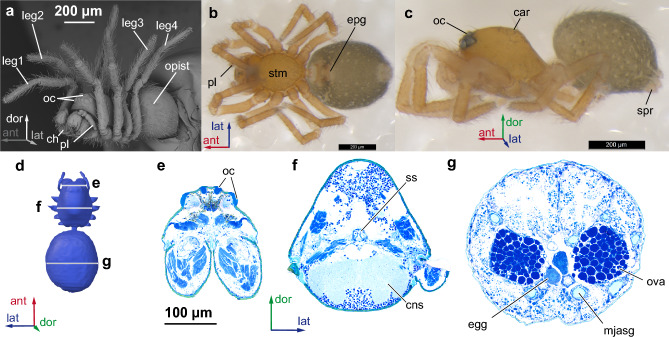
Habitus of *Rayforstia* male, SEM, lateral view (**a**), female, light microscopy, ventral (**b**), lateral (**c**) view, scheme of histological slices top view (**d**), histological sections of the spider *Rayforstia* at different levels (**e**–**g**); ant, anterior; dor, dorsal; lat, lateral; car, carapace; ch, chelicera; cns, central nervous system; egg, egg; epg, epigyne; leg1–4, legs; mjasg, major ampullate silk glands; oc, eyes; opist, opisthosoma; ova, ovary; pl, palps; spr, spinnerets; ss, sucking stomach; stm, sternum. 3D models were processed using Blender 2.93.6 program (https://www.blender.org/download/lts/2-93/).

**Figure 2:**
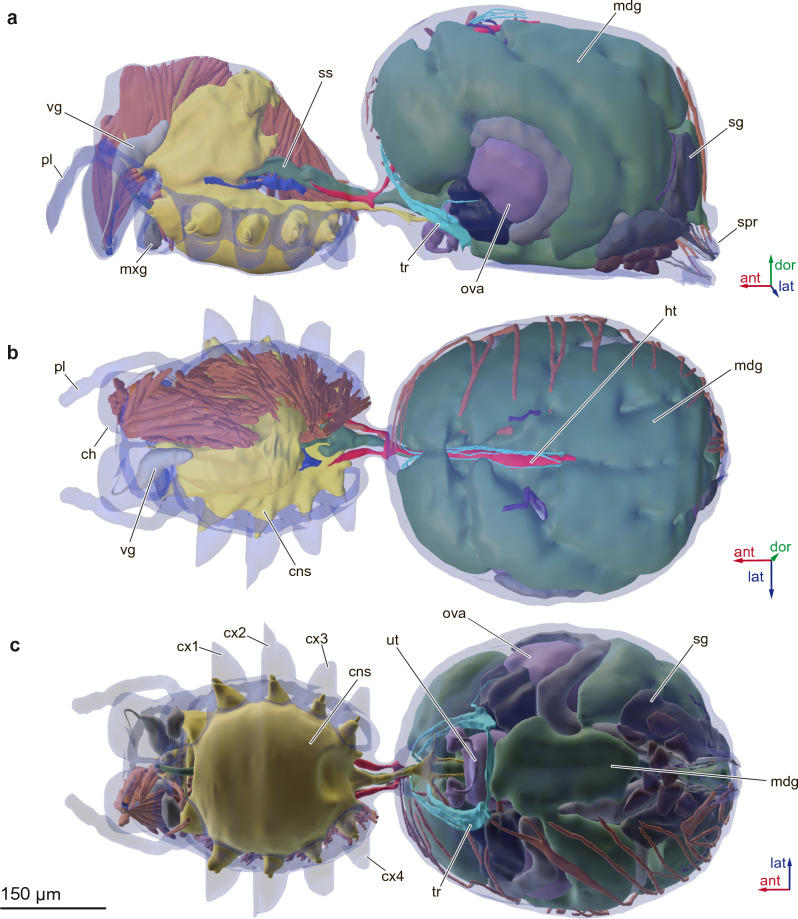
Anatomy of the female spider *Rayforstia* 3D reconstruction (**a**-**c**), lateral view (**a**), dorsal view (**b**), ventral view (**c**); ant, anterior; dor, dorsal; lat, lateral; ch, chelicera; cns, central nervous system; cx1–4, coxa 1–4; ht, heart; mdg, midgut gland; mxg, maxillary gland; ova, ovary; pl, palps; sg, silk glands; spr, spinnerets; ss, sucking stomach; tr, trachea; ut, uterus; vg, venom gland. 3D models were processed using Blender 2.93.6 program (https://www.blender.org/download/lts/2-93/).

Average body length 867 µm (n = 4, three females and one male). General anatomy typical for spiders: body divided into prosoma and opisthosoma, four pairs of walking legs, eight eyes (Fig. 1). Dorsal abdominal scute absent. Opisthosoma behind pedicel with genital opening covered by epigyne. Six spinnerets situated posterior to fleshy colulus. Most of prosoma occupied by brain, most of opisthosoma is occupied by midgut gland and silk glands (Fig. 2). Musculature well-developed in both prosoma and opisthosoma.

Body volume is 59.6 nL.

### Cuticle

Cuticle thickness 1.9–8.8 µm (n = 80). Cuticle thickness 2.0–8.8 µm (n = 32) in prosoma. Cuticle thickness in opisthosoma 1.9–8.3 µm (n = 48).

**Figure 3:**
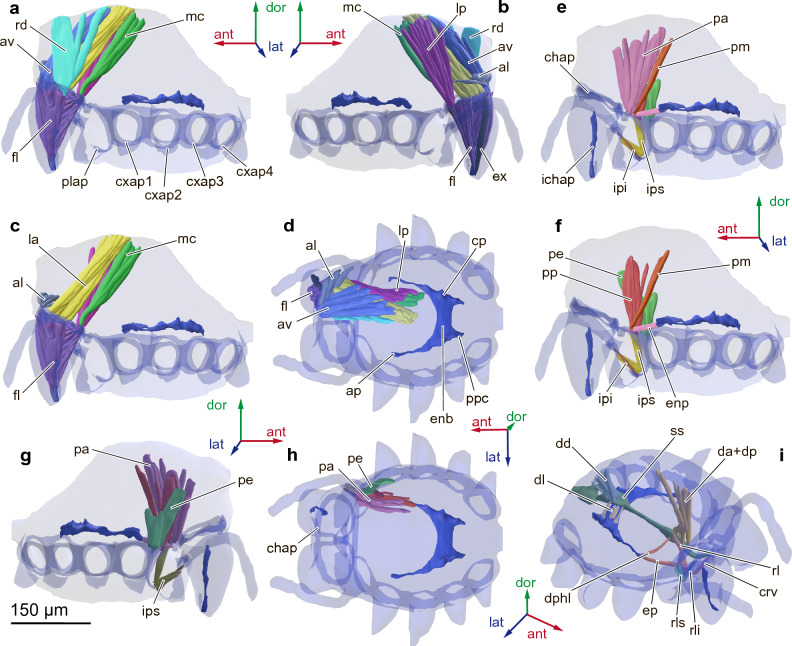
Cheliceral (**a**-**d**) and palpal (**e**–**h**) muscles and muscles associated with the digestive system (**i**) of the spider *Rayforstia*, 3D reconstruction, lateral internal view (**a**, **c**, **e**, **f**), the left side of the cuticle is removed, lateral external view (**b**, **g**), dorsal view (**d**, **h**), three-quarter view (**i**); ant, anterior; dor, dorsal; lat, lateral; ap, anterior process of endosternite; chap, cheliceral apodemes; cp, central process of endosternite; cxap1–4, coxal apodemes 1–4; enb, endosternite body; ichap, intercheliceral apodemes; plap, palpal apodemes; ppc, posterior process of endosternite; ss, sucking stomach. For the nomenclature of the musculature, see Table 1. 3D models were processed using Blender 2.93.6 program (https://www.blender.org/download/lts/2-93/).

Unpaired tergal apodeme absent, endosternite of thorax well-developed, U-shaped, behind CNS forming one plate — body of endosternite (Fig. 3d). Two anterior processes branching arterial. Pair of triangular central processes of endosternite and pair of triangular posterior process of endosternite extending dorsal. Bases of chelicera, palps and walking legs containing apodemes needed for mobility of mouthparts and legs. Basal segment of chelicera medially with elongated cylindrical internal cheliceral apodeme (Fig. 3e).

No opisthosomal endosternites observed.

Volume of exoskeleton 4.7 nL (7.9% of body volume).

### Muscles

**Figure 4:**
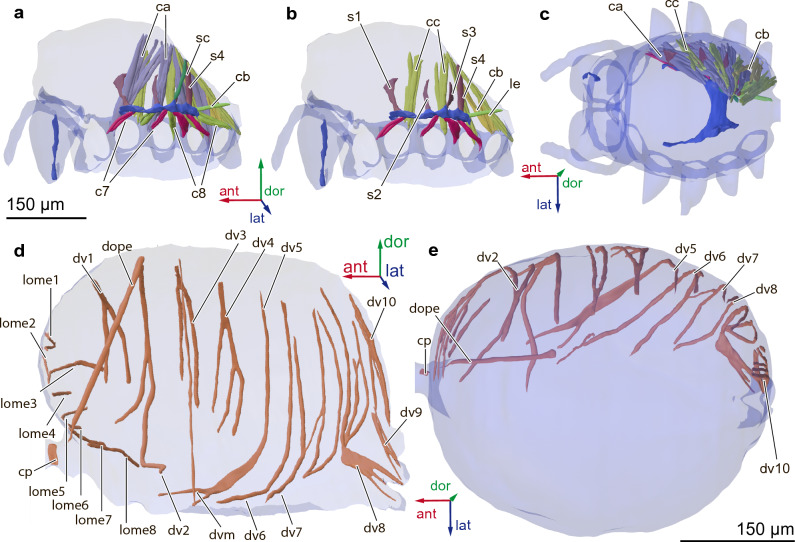
Endosternite, leg muscles (**a**–**c**), and muscles of the opisthosoma and pedicel (**d**, **e**) of the spider *Rayforstia*, 3D reconstruction, lateral internal view (**a**, **b**, **d**), the left side of the cuticle is removed, part of the muscle removed (**b**), dorsal view (**c**, **e**); ant, anterior; dor, dorsal; lat, lateral. For the nomenclature of the musculature, see Table 1. 3D models were processed using Blender 2.93.6 program (https://www.blender.org/download/lts/2-93/).

Prosoma without leg muscles contains 44 pairs of muscles and 3 unpaired muscles (total 89 muscles, Table 1): 8 pairs of cheliceral muscles (Fig. 3a–d), 7 pairs of palp muscles (Fig. 3e–h), 2 paired and 1 unpaired labrum muscles, 3 paired and 2 unpaired muscles associated with digestive system (Fig. 3i), 17 pairs of coxal muscles and 7 pairs of endosternite muscles (Fig. 4a–c). Each leg contains 25 muscles (Table 1).

**Table 1:** Muscles of *Rayforstia* sp.

Abbr	Name of muscle	Origin	Insertion
*Cheliceral muscles (*Fig. 3* a-d)*			
al	m. antero-medialis lateralis	Antero-distal part of the carapace	Anterior part of the cheliceral apodeme
av	m. antero-medialis verticalis	Antero-lateral part of the carapace	Antero-medial part of the cheliceral apodeme
rd	m. medial. retro-desccndens	Antero-medial part of the carapace	Medial part of the cheliceral apodeme
ex	m. cheliceral extensor	Ventro-lateral part of the cheliceral apodeme	Base of the chelicera fang
fl	m. cheliceral flexor	Ventral part of the cheliceral apodeme	Internal cheliceral apodeme
la	m. lateralis anterior	Medial part of the carapace	Lateral part of the cheliceral apodeme
lp	m. lateralis posterior	Lateral part of the carapace	Postero-lateral part of the cheliceral apodeme
mc	m. postero-medial carapacis	Lateral part of the carapace	Posterior part of the cheliceral apodeme
* Palpal muscles (*Fig. 3* e–h)*			
enp	m. endosterno-palparis	Anterior process of the endosternite	Dorsal part of the plap
pa	m. tergo-palpalis anterior	Lateral part of the carapace	Dorso-medial part of the palp coxa
pe	m. tergo-palpalis externus	Lateral part of the carapace	Dorso-lateral part of the palp coxa
pm	m. tergo-palpalis medius	Lateral part of the carapace	Dorsal part of the palp coxa, between pa and pe
pp	m. tergo-palpalis posterior	Lateral part of the carapace	Lateral part of the palp coxa
ips	m. inter-palpis superior	Ventral part of the palpal apodeme	Dorsal part of the palpal apodeme
ipi	m. inter-palpis inferior	Ventral part of the palpal apodeme	Palp trochanter
*Muscles of labrum (*Fig. 3 i*)*			
crv	m. compressor rostri ventralis	Lateral part of the labrum	Lateral part of the labrum
rls	m. rostri lateralis superior	Ventral part of the labrum	Dorsal part of the palpal apodeme
rli	m. rostri lateralis inferior	Ventral part of the labrum	Dorsal part of the labrum
*Muscles associated with digestive system (*Fig. 3 i*)*			
da + dp	m. dilator pharyngis anterior + posterior	Medial part of the carapace	Dorsal part of the pharynx
dd	m. dilator proventriculi dorsalis	Medial part of the carapace	Dorsal part of the sucking stomach
dl	m. dilator proventriculi lateralis	Endosternite body	Lateral part of the sucking stomach
dphl	m. lateral pharynx dilator	Anterior process of endosternite	Lateral part of the pharynx
rl	m. retractor labialis	Ventral part of the labrum	Ventral part of the pharynx
*Endosternite, coxal muscles (*Fig. 4* a-c)*			
ca (1–4)	m. tergo-coxalis anterior	Lateral part of the carapace	Anterior part of the coxa
cb (4)	m. tergo-coxalis medius communis (anterior + posterior)	Lateral part of the carapace	Medial part of the coxa
cc (1–4)	m. tergo-coxalis posterior	Lateral part of the carapace	Posterior part of the coxa
c7 (1–4)	m. endosterno-coxalis anterior	Lateral part of the endosternite	Anterior part of the coxa
c8 (1–4)	m. endosterno-coxalis posterior	Lateral part of the endosternite	Posterior part of the coxa
ep	m. protractor endosterni	Anterior process of endosternite	Dorsal part of the labrum
le	m. loro-endosternalis	Posterior process of endosternite	Anterior part of the pedicel
sc	m. suspensor centralis	Central process of endosternite	Posterior part of the carapace
s1–4	suspensor endosterni I–IV	Lateral part of the endosternite	Lateral part of the carapace
*Muscles of opisthosoma and pedicel (*Fig. 4* d-e)*			
cp	m. pedicel compressor	Dorsal part of the pedicel	Ventral part of the pedicel
dope	m. dorso-pedicel	Dorsal part of the opisthosoma	Posterior part of the pedicel
dvm	m. dorso-ventral medial	Dorsal part of the opisthosoma	Ventral part of the opisthosoma
dv1–10	m. dorso-ventral I–X	Dorsal part of the opisthosoma	Ventral part of the opisthosoma
lome1–8	m. lateromedial muscles I–IIX	Lateral part of the opisthosoma	Medial part of the opisthosoma
*Leg muscles (not shown on the figures)*			
	m. levator pretarsi (paired)	Dorsal proximal part of the metatarsus	Claw
	m. depressor pretarsi	Ventral proximal part of the metatarsus	Claw
	m. flexor tarsi anticus	Proximal part of the tibia	Proximal part of the metatarsus
	m. flexor tarsi posticus	Proximal part of the tibia	Proximal part of the metatarsus
	m. flexor tarsi major	Medial part of the tibia	Proximal part of the metatarsus
	m. flexor tarsi minor	Medial part of the tibia	Proximal part of the metatarsus
	m. promoter tibiae	Dorsal part of the patella	Proximal part of the tibia
	m. remotor tibiae	Dorsal part of the patella	Proximal part of the tibia
	m. flexor patellae robustus (paired)	Dorsal proximal part of the femur	Dorsal distal part of the femur
	m. flexor patellae major (anterior)	Dorsal distal part of the femur	Proximal part of the patella
	m. flexor patellae minor (posterior)	Dorsal distal part of the femur	Proximal part of the patella
	m. extensor femoris posticus	Proximal part of the femur	Median part of the femur
	m. extensor femoris proximalis	Proximal part of the femur	Median part of the femur
	m. extensor femoris princeps	Proximal part of the femur	Median part of the femur
	m. extensor femoris communis	Proximal part of the femur	Median part of the femur
	m. flexor femoris bilobatus	Ventral part of the trochanter	Dorsal proximal part of the femur
	m. gracilis	Dorsal part of the trochanter	Dorsal proximal part of the femur
	m. promotor trochanteris	Lateral part of the coxa	Proximal part of the trochanter
	m. remotor trochanteris ventralis	Ventral part of the coxa	Proximal part of the trochanter
	m. remotor trochanteris dorsalis	Dorsal part of the coxa	Proximal part of the trochanter
	m. depressor trochanteris obliquus	Proximal part of the coxa	Proximal part of the trochanter
	m. depressor trochanteris ventralis	Ventral part of the coxa	Proximal part of the trochanter
	m. depressor trochanteris medius	Coxal apodeme	Ventral proximal part of the trochanter
	m. levator trochanteris anticus	Coxal apodeme	Proximal part of the trochanter
	m. levator trochanteris posticus	Dorsal part of the coxa	Proximal part of the trochanter

Pedicel containing one pair of compressor muscles.

Opisthosoma containing 20 pairs of muscles, dorsoventral and lateromedial (Fig. 4d, e).

Muscle volume without leg muscles 1.4 nL (2.3% of body volume), most muscles (0.6 nL) situated in prosoma (3.6% of its volume).

### Central nervous system

**Figure 5:**
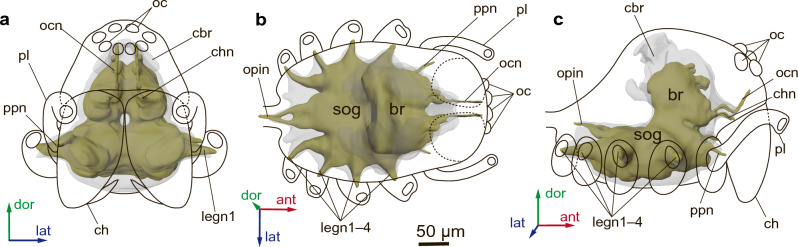
Central nervous system of the spider *Rayforstia*, 3D reconstruction, anterior view (**a**), dorsal view (**b**), lateral view (**c**); ant, anterior; dor, dorsal; lat, lateral. br, brain; cbr, cell body rind; ch, chelicera; chn, cheliceral nerve; legn1–4, leg nerves; oc, eyes; ocn, ocellar nerve; opin, opisthosomal nerve; pl, palps; ppn, palpal nerves; sog, subesophageal ganglion. 3D models were processed using Blender 2.93.6 program (https://www.blender.org/download/lts/2-93/).

CNS represented by large synganglion surrounding esophagus as in most spiders^28–33^ (Fig. 5a–c). In spite of the high degree of cephalization and of concentration of the CNS in spiders, individual ganglia can be recognized by neuromeres. The brain of *Rayforstia* consists of three neuromeres: the ocular neuromere, cheliceral neuromere and palp neuromere. Palp neuromere situated in anterior subesophageal area of the CNS synganglion (Fig. 5a). The subesophageal ganglion consists of fused neuromeres associated with walking legs and opisthosomal ganglia. The brain gives pair of optic, cheliceral and palpal nerves. From subesophageal ganglion arise four pairs of leg nerves, associated with the walking limbs, and pair of opisthosomal nerves (Fig. 5a–c), which goes into the opisthosoma, where it gives rise to two more pairs of lateral nerves leading to silk glands and spermatheca.

In neuropil of *Rayforstia* brain arcuate body, cheliceral and palps neuropils well identified. Mushroom bodies and visual neuropils due to small size and low resolution of light microscopy weakly discern. Arcuate body well differentiating crescent shaped neuropil located at posterior part of brain composed of distal and ventral divisions (Fig. S2), have an average volume about 0.05 nL and occupy up to 1% of the CNS volume (and about 3% of brain volume). Visual neuropils, mushroom bodies, palps and chelicerae neuropils occupy each not far then 1% of total brain volume.

CNS volume 5.3 nL (9.0% of body volume and 31.5% of prosoma volume). Brain volume 2.1 nL (3.5% of the body volume, 12.5% of prosoma volume). CNS contains about 34 700 neurons; around 19 800 of them belong to brain. Average diameter of cell bodies in CNS 3.9 ± 0.30 μm.

### Digestive system

**Figure 6:**
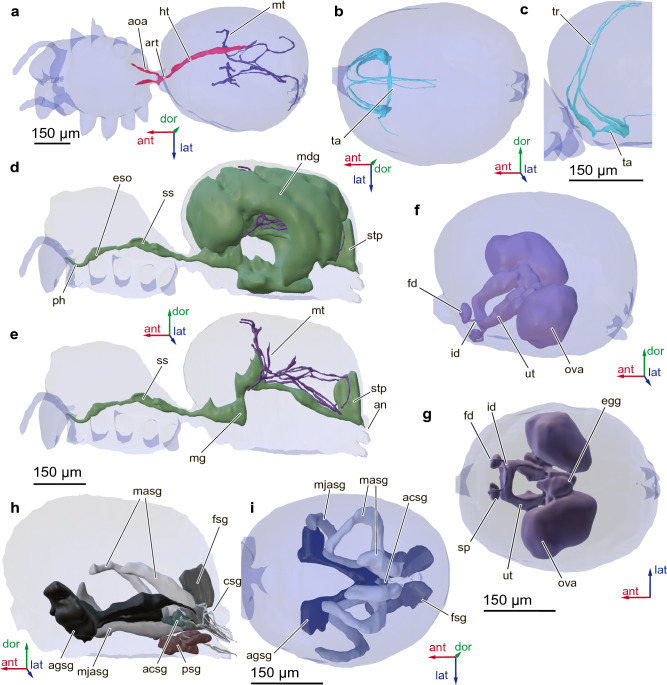
Circulatory and excretory (**a**, **d**, **e**), respiratory (**b**, **c**), digestive (**d**, **e**), reproductive (**f**, **g**) systems and glands (**h**, **i**) of the spider *Rayforstia*, 3D reconstruction, dorsal view (**a**, **b**, **i**), lateral internal view, the left side of the cuticle is removed, part of glands is removed (**d**, **e**, **h**), lateral view (**c**), three-quarter view (**f**), ventral view (**g**); ant, anterior; dor, dorsal; lat, lateral; acsg, aciniform silk glands; agsg, aggregate silk glands; an, anus; aoa, aortic arches; art, aorta; csg, cylindrical silk glands, egg, egg; eso, esophagus; fd, fertilization duct; fsg, flagelliform silk glands; ht, heart; id, insemination duct; masg, minor ampullate silk glands; mg, midgut; mdg, midgut gland; mjasg, major ampullate silk glands; mt, malpighian tubules; ova, ovary; ph, pharynx; psg, piriform silk glands; sp, spermatheca; stp, stercoral pocket; ss, sucking stomach; ta, tracheal atrium; tr, trachea; ut, uterus. 3D models were processed using Blender 2.93.6 program (https://www.blender.org/download/lts/2-93/).

Digestive system divided into the foregut, midgut and hindgut (Fig. 6d, e). Pharynx and esophagus (about 10.4 µm in diameter, 106 µm long), which passes through CNS, originate from mouth opening. Esophagus then passes into sucking stomach. Sucking stomach about 29.5 µm high and 84.2 µm long, shaped as collapsed square. Midgut almost not branched in prosoma. Two small outgrowths 89.0 µm long extend arterial from sucking stomach. Most of the volume of opisthosoma occupied by outgrowth of midgut, midgut gland. Posterior part of midgut forms extension, in spiders termed stercoral pocket (height 117 µm), followed by hindgut ending in anus.

Digestive system volume 18.7 nL (31.4% of body volume), midgut gland volume 16.7 nL.

### Reproductive system

Epigyne poorly sclerotized, with pair of copulatory openings placed in front of epigastric furrow. Female reproductive system situated in ventral part of abdomen and includes ‘bean-shaped’ spermathecae, short fertilization ducts, long and coiled copulatory ducts, uterus, ovaries and eggs coming out of them (Fig. 6f, g). Spermathecae oval, length 45.4 µm, width 22.7 µm. Fertilization ducts short and coiled, adjoining spermathecae in front, length 34.3 µm, width 2.8 µm. Insemination ducts curved, C-shaped, 54.7 µm long, 8.0 µm wide. Uterus U-shaped, situated beneath digestive system, its outgrowths wrapping around midgut, 89.4 µm wide, 78.8 µm high. Rounded eggs, with an average diameter 37.0 µm, situated behind uterus. Paired ovaries situated lateral of eggs, oval in shape, 130 µm wide, 191 µm long. Ovaries occupying most of reproductive system, their volume 3.4 nL.

Reproductive system volume 4.0 nL (6.8% of body volume).

### Circulatory system

Circulatory system includes heart, about 17.8 µm in diameter, 285 µm long, located on dorsal side of opisthosoma above midgut gland, aorta 57.7 µm long, passing through stalk into the prosoma, and two cylindrical aortic arches 73.6 µm long, 14.1 µm in diameter, running laterally from midgut (Fig. 6a). Heart has appearance of an elongated tube. There is almost no aortic branching in prosoma, no head artery and no leg arteries.

Space between internal organs filled with connective tissue, similar to fat body of insects^35^. It surrounds internal organs as thin membranes represented by small (5.0–10.0 µm) cells, which may contain various inclusions.

Circulatory system and connective tissue volume 21.0 nL (35.2% of body volume).

### Respiratory system

Respiratory system includes transverse atrium 111 µm long, located ventrally in opisthosoma behind genital opening, and tracheae branching from atrium (Fig. 6b, c). There are six tracheae, three on each side, curved dorsally from the anterior to posterior end of opisthosoma and not extended into the prosoma.

Anterior tracheal spiracles present on either side of the epigastric furrow, leading to the anterior tracheae.

Book lungs absent.

Respiratory system volume 0.06 nL (0.1% of body volume).

### Glands

Venom glands elongated, cylindrical (Fig. 1a, b), located in anterior part of prosoma, their adjoining duct going to chelicera and subapically on cheliceral fang. Average length of ampulla of venom gland 122 µm. Average diameter of venom gland ampulla 39.7 µm. Average adjoining duct length 110 µm.

Venom gland volume 0.2 nL (0.3% of body volume).

Maxillary glands oval in shape, located in coxae of palps, their ducts opening on inner surface of coxae (Fig. 1a). Average diameter of widest part of gland 37.9 µm. Duct length 7.6 µm.

Maxillary gland volume 0.05 nL (0.08% of body volume).

Seven types of silk glands present: pair of major ampullate glands, 10 piriform glands, two pairs of minor ampullate glands, two pairs of flagelliform glands, pair of aggregate glands, three pairs of aciniform glands, and two pairs of cylindrical glands (Fig. 6h, i).

Major ampullate glands elongate, extending to anterior end of opisthosoma and curving dorsally on sides of ovary, their ducts opening on anterior spinnerets. Average length of major ampullate glands 301 µm, width about 39.9 µm. Piriform glands oval and round in shape in lower part of opisthosoma, close to spinnerets, their ducts opening on anterior spinnerets. Average diameter of piriform glands 25.7 µm. Minor ampullate glands also elongate, anterior pair laterally bordering ovary, posterior pair shorter, adjoining dorsal part of ovary, their ducts opening on middle spinnerets. Length of anterior minor ampullate glands 371 µm, width 40.8. Length of posterior minor ampullate glands 210 µm, width 34.2. Flagelliform glands oval, anterior pair adjoining ventral opisthosomal part, posterior pair adjoining caudal ventral part, their ducts opening on posterior spinnerets, height 116 µm, width 58.7 µm. Aggregate glands large, most of each gland’s body located anterior to ovary, their ducts opening on posterior spinnerets. Width of anterior part of gland body (in front of ovary) 89.3 μm, width of extended part of ampulla 26.4 μm, length 254 μm. Aciniform glands small, oval, located between minor ampullate glands, their ducts opening on posterior spinnerets, length 34.5 µm, width 21.4 µm. Cylindrical glands thin and elongated, tightly adherent to the walls of the minor ampullate glands. Length of cylindrical glands is about 110 µm, width 4–5 µm.

Volume of silk glands 4.2 nL (7.0% of body volume). Volumes of gland types: major ampullate glands 0.79 nL, piriform glands 0.14 nL, minor ampullate glands 1.3 nL, flagelliform glands 1.0 nL, aggregate glands 0.9 nL, aciniform glands 0.015 nL, cylindrical glands 0.007 nL.

### Excretory system

Excretory system represented by paired, branched Malpighian tubules (Fig. 6a, e). They open in anterior part of stercoral pocket; their branches extend into anterior and posterior parts of abdomen and do not enter prosoma. Average diameter of main tubules about 9.2 µm.

Malpighian tubules volume 0.1 nL (0.2% of body volume).

## Discussion

### General anatomy and cuticle

The protruding sternum of the prosoma we observed in *Rayforstia* has been reported previously for Anapidae: e.g., it is characteristic of *Tasmanapis strahan* and *Anapisona simoni* . It is hypothesized to be a result of reaching the limits of the CNS miniaturization in spiders allowing to get the extra space for the relatively large brain^25^.

*Rayforstia* lacks the tergal apodeme to which dorsal muscles of the sucking stomach are attached. This is characteristic of many species of the family^27^.

### Muscles

A total of 67 pairs of muscles and three unpaired ones were described for the prosoma of Araneomorphae^34,35^; two more muscles, anterior outer and inter-cheliceral-sclerite muscles, were later described using computed tomography in palpimanoid spiders^13^. The exact numbers of muscles in different families are difficult to operate with, as Palmgren^34^ did not count them, and many are not indicated in figures. For *Rayforstia*, we described five other pairs: m. endosterno-palparis, m. inter-palpis superior, m. inter-palpis inferior, m. rostri lateralis superior, m. rostri lateralis inferior.

The most conserved muscle group is the cheliceral muscles: in almost all spiders their set remains the same, and the number of leg muscles changes the most (Table S1). Combining our data and data on prosomal muscles of Araneoidea according to Palmgren^34^, we conclude that in spiders there is no dependence of the amount of muscle on body size, both in direct counting of all the muscles indicated in the figures, and when all muscles not shown in figures in Palmgren publication (marked "?" in Table S1) are considered. Slopes according phylogenetic regression analysis are 0.03 and 0.05, respectively and not significantly differ from zero, p = 0.5 and 0.3. But in the smallest studied spider the number of muscles is distinctly smaller than in most large spiders and comparable with smallest value in large spiders.

Some species, among them *Comaroma simoni* (Anapidae), body length 1.6 mm, have a muscle configuration very different from the large Araneoidea, such as *Larinioides cornutus* (6.0–20.0 mm, Araneidae)^35^. In particular, *C. simoni* lacks individual c1–c6 muscles (m. tergo-coxalis anterior profundus, m. t-c medius anterior, m. t-c medius posterior, m. t-c posterior profundus, m. endosterno-coxalis antero-superior, m. e-c postero-superior). A unique feature of *C. simoni* is the muscles ca, cb, cc (m. t-c anterior, m. t-c medius communis and m. t-c posterior), which we also describe in *Rayforstia*. Moreover, in *Rayforstia* cb is found only in leg pair IV, suggesting further reduction of leg muscles with decreasing size. However, the smaller spider *Minyriolus pusillus* (Linyphiidae, 1.1–1.4 mm) retains the complete set of leg muscles, in contrast to *C. simoni*^34^. Apparently, the ca, cb, cc muscles are a common feature of symphytognathoid spiders, but are not necessarily related to miniaturization, as they are not characteristic of the small Theridiidae (*Parasteatoda lunata*, *Theridion pictum*)^35^.

In *C. simoni* only one of the musculi laterales, near coxa1, was found; in *Rayforstia* these muscles were not found at all.

In *C. simoni* two m. dilator pharyngis are found, while in *Rayforstia* they merge into one (da + dp). This fusion is observed in spiders of different sizes (Table S1. The m. medialis pro-descendens is absent from the cheliceral muscles in *Rayforstia* in contrast to *C. simoni*.

Among the muscles of the endosternite, m. plagulo-endosternalis dorsalis and ventralis are absent in *Rayforstia,* in contrast to *C. simoni*. Of the muscles arising from the carapace, m. loro-tergalis is absent.

Compared to the spiders examined by Palmgren^34^, only *Rayforstia* have the pe muscle (m. tergo-palpalis externus).

But not all muscles are reduced in number. In Linyphiidae among the Araneoidea^36,37^, the ed + ev muscle (m. plagulo endosternalis dorsalis and ventralis) has been split into two separate muscles.

At the same time, with a decrease in size, the musculature of the legs almost does not change in *Rayforstia*. Compared to larger *Avicularia* (Theraphosidae)^38^ and *Tegenaria atrica* (Agelenidae)^39^, *Rayforstia* lacks muscles flexor patellae brevis, m. depressor tibiae anticus and posticus and has an unpaired m. depressor pretarsi.

In contrast to larger spiders, *Liphistius* (Liphistiidae) and *Araneus ventricosus* (Araneidae)^40^, *Rayforstia* has no longitudinal opisthosomal muscles and generally no muscles on ventral surface of opisthosoma. However, for *Rayforstia* lateromedial muscles are described for the first time.

### Central nervous system

The CNS miniaturization in most arthropods is characterized by several common features. In the smallest spiders^25,26^, as in the smallest mites^22^, there is an increase in the relative volume of the CNS, a strong decrease in the size of the nervous system cells and displacement of the brain or thoracic ganglia into other tagmata. In *Rayforstia,* similarly to other miniature spiders, the presence of CNS extensions into the coxae and palps and a protrusion of the sternum of the prosoma are noted.

In *Rayforstia* the arcuate body occupy up to 3% of the brain volume, that similar with the results on some larger spiders with or without web-weaving behaviour: *Nephila clauipes* (Araneidae) 3,8%, *Phidippus regius* (Salticidae)—3,1%^41^; *Eratigena atrica* (Agelenidae)^42^, thus should not be associate just with particular activity of web-weaving^29,41,42^.

Arcuate body of chelicerates and central body of hexapods demonstrate a large number of similarities in architectural characters and innervation^31^ as navigational control center that integrated visual information^43^. It is interesting that the proportion of the central body volume in the brain volume of insects of various body size and biology is similar and relatively small (1–2%)^44–47^.

The number of cells in the CNS of *Rayforstia* is about 34 700, of which about 19 800 belong to the brain. In large spiders (*Cupiennius salei*), the CNS has about 100 000 cells, of which 50 890 belong to the brain^29^.

The cell size of *Rayforstia* CNS averages 3.9 µm, which is smaller than in large spiders, such as *Cupiennius salei* (Trechaleidae), 12.0–20.0 µm^29^, but larger than in miniature mites: 1.8 µm in *Phytoseiulus persimilis* (Parasitiformes: Phytoseiidae)^48^ and 1.1 µm in *Achaetocoptes quercifolii* (Acariformes: Eriophyoidea)^22^.

The CNS volume in *Rayforstia* is 9.0% relative to body volume (prosoma + opisthosoma) and 31% relative to prosoma, which is more than 7 times greater than in large spiders, about 4% in late juvenile instars and adults^49,50^, and is consistent with published data on other miniature spiders^26^.

The relative neuropilar volume (RNV) of the brain in arachnids (as in insects) increases during development from juveniles (instars or larvae) to adults^26,42,49–51^ due to growth and differentiation of neuropilar centres. However, RNV in most adult insects is constant at about 60%^52^, regardless of body size. It can be speculated that in spiders the rule of neuropilar constant is also conserved. The RNV is 75% in adults in a large spider, *Argiope aurantia* (Araneidae)^49^, 66% in late juvenile instars of *Eratigena atrica* (Agelenidae)^42^ and 62% in *Rayforstia*. Interestingly, in *A. aurantia* the RNV is slightly higher than the constant, which may be due to the method of volume measurement used in older studies.

Similar to microinsects, which despite their tiny brains retain associative learning and memory capabilities^18,53^, minute spiders also seem to retain behaviours similar to those of larger ones^54^. Various studies on brain size and the complexity of orb web-making behaviour show that smaller spiders with smaller brains are not constrained in behaviour and can make webs identical to those of larger ones, without any structural differences^17,25,55^. Furthermore, it has been shown that there is no difference between webs made by juveniles and adults^56^. Despite their small size, miniature spiders perform complex sets of actions when weaving webs^17,54^. However, in a study on the memory of small spiders, it was found that when searching for lost prey, they forget where they left it sooner than larger ones^57^.

### Digestive system

The digestive system of *Rayforstia* is distinguished by the absence of branching of the midgut in the prosoma, probably due to the need for additional space for the brain. This has previously been reported only for juvenile instars of *Phidippus clarus* (Salticidae), but not for adult spiders^25^. The relative volume of digestive system in *Rayforstia* is 31.4%, which is higher than in miniature mites *A. quercifolii* (0.054%)^22^ or insects: 21% in *Liposcelis* (Insecta: Psocodea: Liposcelididae))^58^.

### Reproductive system

The reproductive system of a miniature spider is not dramatically different from that of larger spiders^9^. This is also generally the case for other small arthropods, although almost all featherwing beetles and four-legged mites have unpaired gonads^19,22^. The relative volume of the reproductive system is much lower than in other microarthropods: 6.8% in *Rayforstia*, 34.8% in the four-legged mite *A. quercifolii*^22^, 12.8% in *Liposcelis*, 22.7% in *Sericoderus* (Insecta: Coleoptera: Corylophidae) and 12.3% *Mikado* (Coleoptera: Ptiliidae)^58^.

### Circulatory system

In contrast to the larger *Araneus diadematus* (Araneidae)^9,59^, in *Rayforstia* the circulatory system is simplified: the aorta in the prosoma divides into only two aortic arches and does not branch into the artery systems. There is also no branching of the cardiac arteries. In some minute insects (Ptiliidae and Trichogrammatidae) and mites (Eriophyoidea), the circulatory system is absent; in other minute insects it is simplified, consisting of only the heart and aorta^19,22^, as in *Rayforstia*.

### Respiratory system

The respiratory variation found in the studied spider is typical of *Rayforstia*^10,27^: posterior spiracles and book lungs are absent; only anterior spiracles, one on each side of the epigastric furrow, are present. Anapids as a whole are characterized by large variations in the structure of the respiratory system^10^, anterior tracheae transformed to book lungs and reductions multiple times and posterior tracheae were lost multiple times^12^. It is highly unlikely that this structure of the respiratory system of *Rayforstia* is a consequence of miniaturization since in different genera of approximately the same size (1–2 mm), a significant variability of the respiratory systems is observed^10^.

The respiratory system is absent in some miniature mites and collembolans^21,22^, and in microinsects it is strongly simplified, reduced to longitudinal stems^19^, but in *Rayforstia* it remains complex.

### Glands

In the studied spider, in comparison with the larger *Araneus diadematus* (Araneidae)^40^, *Nephila* (Araneidae)^60^ the number of glands is decreased: from three to two pairs of cylindrical glands, from two to one pair of aggregate glands, the number of piriform glands is 10, which is much smaller than in *A. diadematus* (100).

The number of aciniform gland spigots among symphytognathids varies from zero to three^27^, and there is a general tendency towards reduction in the number of aciniform gland spigots in the subfamily Micropholcommatinae.

### Excretory system

As in many spiders^9,40^, the main organs of excretion in *Rayforstia* are the Malpighian tubules and stercoral pocket. There is one pair of Malpighian tubules and this number is conserved in almost all spiders^61^. No coxal glands were found as simplification of coxal glands is characteristic of Araneoidea^40^, which includes Anapidae^36,37^.

### Comparison of miniaturization of terrestrial arthropods

The vast majority of spiders are active predators, using webs to hunt. All miniature spiders, including the smallest *Patu digua* (370 µm), are also predators can also produce webs^3^. Consequently, they retain the silk glands (which occupy 7% of their body volume), digestive system, and CNS. Many authors^6,17,26^ noted that the brain size needed for hunting and web-making probably limits the body size of spiders. Apparently, due to the retention of a large brain, symphytognathoids (Symphytognathidae, Mysmenidae and Anapidae) are capable to weave their webs even faster and more precisely than large spiders^17^. Spiders have more options than insects for the CNS and brain placement because of the large prosoma and ability to partially displace the CNS into the coxae of the legs. At the same time, much of the prosoma is occupied by muscles (3.6% of the prosoma volume).

Several trends in the miniaturization of arthropods can be distinguished: reduction in the number and volume of muscles, increase in the relative volume of the CNS and reproductive system and simplification of the digestive, circulatory and excretory systems.

Spiders are rather similar in the ratios of organ volumes to non-flying miniaturized insects and collembolans. In particular, the non-flying *Liposcelis* sized 0.9 mm has a rather large relative volume of the digestive system (21.1%)^58^, in *Megalothorax* sized 0.2 mm (Collembola: Neelipleona: Neelidae) the relative volume of the digestive system is also rather large: 19.8% [Irina Panina, pers. comm.].

However, the relative CNS volume of *Rayforstia* (9%) is closer to those of microinsect larvae than of adults: *Sericoderus* adult 5.5%, larva 9%; *Mikado* adult 6.7%, larva 16%^58^. The miniaturization of insects is limited mainly by two factors^19^: the volume of the CNS (the minimum size of which is limited by the number of neurons and their minimal size), and (in non-parasitic species) by the reproductive system (the minimum size of which is limited by the egg volume). For the spiders, the volume of the digestive system and silk glands, unique to spiders, appears to be an additional limiting factor.

In general, the internal structure of miniature spiders differs much less from the anatomy of large representatives. There is no reduction of organ systems or a significant increase in the relative volume of the reproductive system as in mites and microinsects. This indicates a significant conservatism in the anatomy of spiders.

## Materials and methods

### Material

The spiders *Rayforstia* cf. *raveni* Rix & Harvey, 2010 were collected by K.Y. Eskov on December 4, 2015, in Tasmania, St. Clair National Park, S 42°07′00.9″, E 146°07′40.1″ and fixed in 70% ethanol (EtOH). Four specimens were examined: one female by histology and 3D reconstruction, one female by confocal microscopy, one female by light microscopy and one male by SEM.

### Histology

Material from 70% ethanol was placed for 12 h in Bouin alcohol and then washed in 70% ethanol. The fixed material was dehydrated in a series of EtOH solutions (in 96% for 30 min and twice in 100% for 30 min) and then in acetone (twice in 100% for 30 min). The material was then placed in Araldite M: incubated in a 1 : 3 mixture of Araldite and acetone for 3 h, then 1 : 1 overnight, then 24 h in a new 1 : 1 mixture of Araldite and acetone in open container, then transferred into pure Araldite for 2 h, changed Araldite and polymerised at 60 °C for two days. The blocks were cut into a series of 1 µm thick cross sections using a Leica RM2255 microtome; the sections were then stained with toluidine blue and mounted in Biomount (Fig. S1).

### CLSM

Spiders from 70% ethanol were fixed in Carnoy's solution for 12 h at 4 °C, then washed in with EtOH 100%, then placed for two days in a solution of dimethyl sulfoxide, ethanol and hydrogen peroxide (DMSO + EtOH + H2O2) at 4 °C. After two days, the solution was changed. After another two days, the material was placed in 70% EtOH for 30 min, in 50% ethanol for 30 min, in 30% ethanol for 30 min, then 0.1 M phosphate buffered saline with 0.5% Triton (PBST) for 5 min, and three times for 30 min, then left for four days in a mixture of 0.01% rhodamine and 3X SYTOX in PBST at 4 °C. The material was then washed with 0.1 M PBST at 4 °C. After 24 h, the material was placed in a clear solution of 0.1 M PBST for 30 min and then the material was kept in ethanol solutions of 30%, 50%, 70%, 95% and twice in 100% ethanol for 30 min each. After that, the material was placed in a mixture of ethanol, benzyl alcohol and benzyl benzoate (BABB) (1 part ethanol : 1 part BABB) for two hours at 4 °C, in BABB solution for two hours at 4 °C and overnight in BABB at 4 °C. One day later, the slide was prepared in BABB and examined under an Olympus FV10i confocal microscope (Fig. S2).

### 3D reconstruction

The slices were photographed using a Motic BA410 c microscope with a Tucsen Michrome 5 pro camera. One adult female was taken and cut into a series of cross-sections (n = 1074). The resulting stacks of photographs were then aligned and calibrated (Fig. A1. 3D reconstruction was carried out with Bitplane Imaris 9.5. In addition, we processed the models using the surface smoothing and rendering functions in Blender 2.93.6.

The volumes of the structures as well as cuticle thicknesses were measured on one female individual using the Bitplane Imaris program.

The body volume was measured without limbs. Leg muscles were not considered for summarising prosoma muscles and calculating muscle volumes, as they were not included in Palmgren^34^ analysis, nor were leg muscles considered in the works on miniature insects. Therefore, comparative analyses were performed without taking leg muscles into account. The CNS volume (directly brain, subesophageal ganglion and their neuropils were measured without nerves (only prosomal nerve mass)).

The thickness of the cuticle was measured on every 10 slices (starting from 10) at 8 opposite points of the cross-section.

### SEM

SEM images were taken on a Tescan Vega2 (Brno, Czech Republic) scanning electron microscopes, operated in a high vacuum mode at the accelerating voltages 10–20 kV, using secondary electron (SE) detector. Specimens were gradually dehydrated in 100% ethanol, dried, and sputter-coated with gold–palladium.

Due to the endless dispute on segmental organization of the arthropod heads^63^, for CNS segmentation we use terminology from the review by Lehmann et al.^64^.

### Nomenclature

The nomenclature proposed by Foelix^9^ and Ivanov^40^ were used to describe the anatomical structures and muscles of the opisthosoma. To unify the terminology, the spider anatomy ontology (SPD) was used^62^. The nomenclature proposed by Palmgren^34^ was used to label the muscles of the prosoma, and for some muscles not described by Palmgren, original names were coined. The nomenclature proposed by Dillon was used to describe the muscles of the legs^38^.

### Muscle number count

Phylogenetic regression analysis in R using the ‘phytools’ package was applied, the phylogenetic tree was constructed from previously published phylogenies^65,66^. The number of spider muscles was taken from Palmgren^34^. Since the author did not specify the exact number of muscles of each species studied, the muscles shown in the figures were counted. Thus, each species has two numbers of muscles: indicated in the figures and the maximum value of the number of muscles, if we assume that those not indicated in the figures are represented.

### Supplementary Information

Supplementary Information 1.

Supplementary Information 2.

## Data Availability

All data analyzed in this study are available in the manuscript and supplementary information file. The original histology and CLSM images are available at https://doi.org/10.5281/zenodo.8219011.
