# Supplementary material for: Miniaturization does not change conserved spider anatomy, a case study on spider Rayforstia (Araneae: Anapidae)
Source: Sci Rep. 2023 Oct 11;13:17219. doi: 10.1038/s41598-023-44230-3 (PMC10567922; doi:10.1038/s41598-023-44230-3)
Supplement: Supplementary file 1 — Supplementary Information 1. [file 41598_2023_44230_MOESM1_ESM.pdf]

Anatomy of the miniature spider *Rayforstia* (Araneae: Anapidae): general principles of arthropod miniaturization vs. conserved spider anatomy

E.A. Propistsova*[1], A.A. Makarova[1], K.Y. Eskov[2], A.A. Polilov[1]

[1] Faculty of Biology, Lomonosov Moscow State University, Moscow, Russia

[2] Borissiak Paleontological Institute of the Russian Academy of Sciences (PIN), Moscow, Russia

1. Figure S1. Anatomy of the spider *Rayforstia*, histological sections at different levels.
2. Figure S2. Anatomy of the spider *Rayforstia*, CLSM images.
3. Table S1. Comparison of the prosomal muscles of *Rayforstia* and some other spiders.

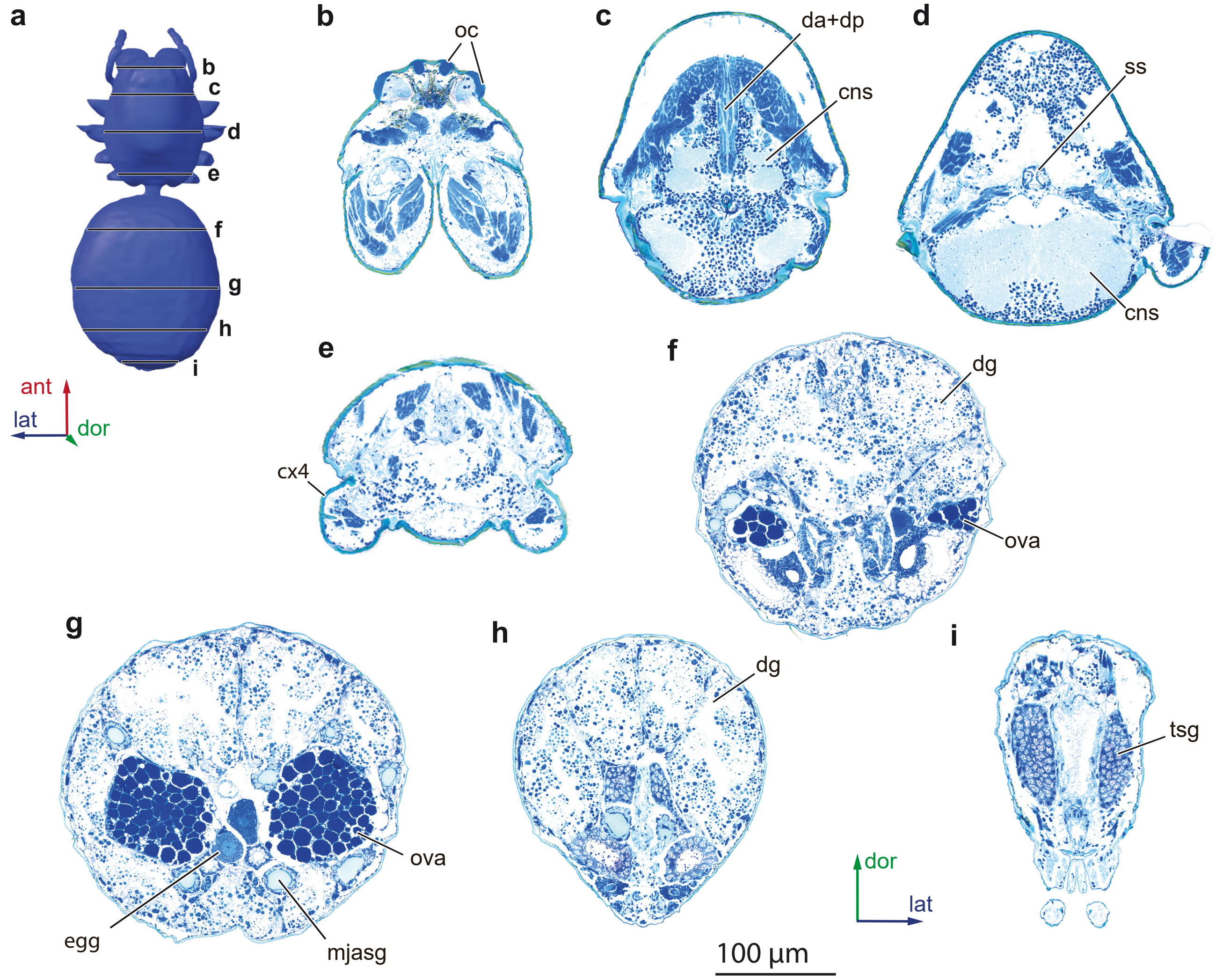

Figure S1. Anatomy of the spider *Rayforstia*, histological sections at different levels. Scheme of slices top view (a), cross sections of the female (b–i); ant, anterior; dor, dorsal; lat, lateral. cns, central nervous system; cx4, coxa 4; da+dp, m. dilator pharyngis anterior+posterior; dg, digestive gland; egg, egg; mjasg, major ampullate silk glands; oc, eyes; ova, ovary; ss, sucking stomach; tsg, tubuliform silk glands.

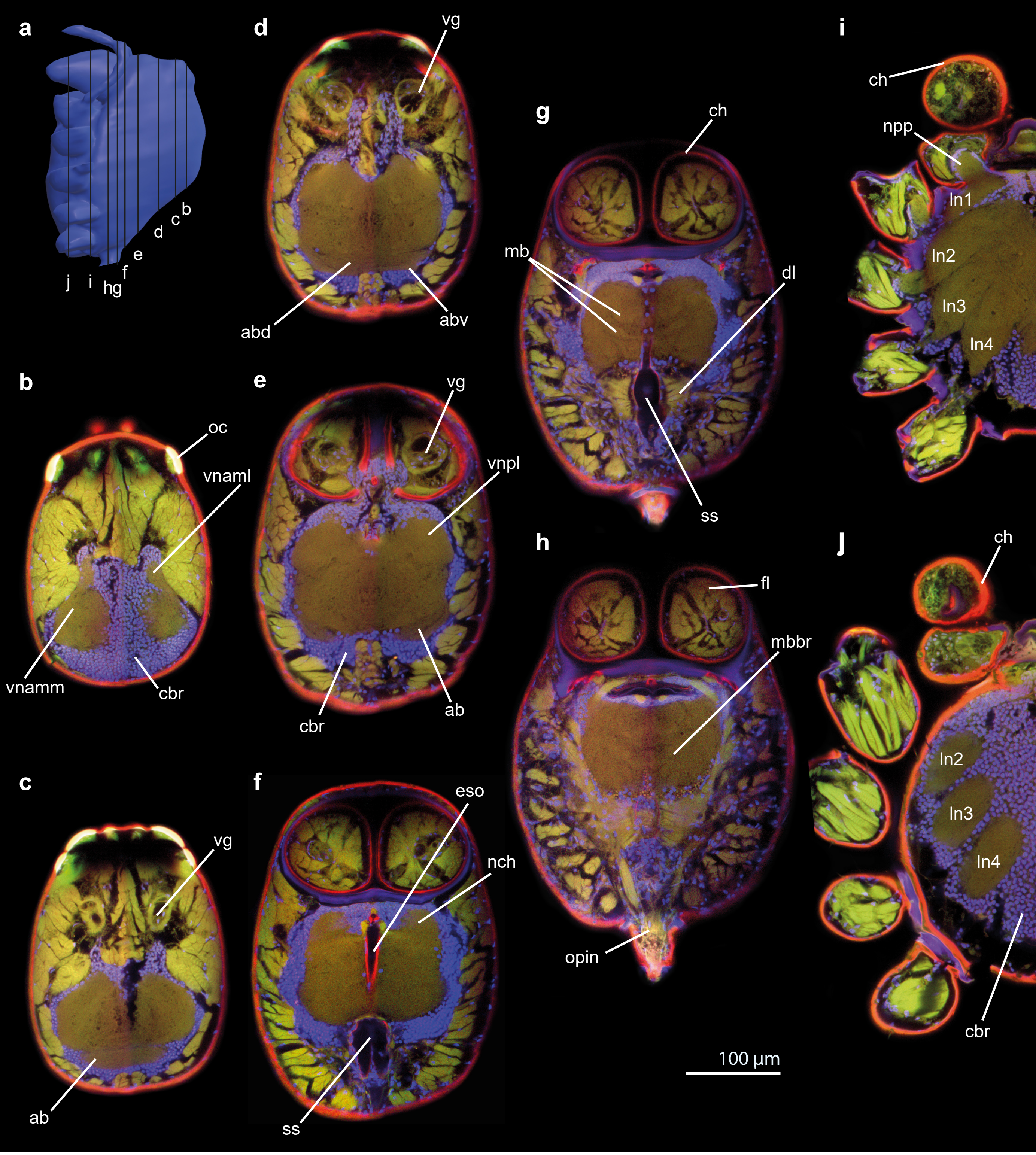

Figure S2. Anatomy of the spider *Rayforstia*, confocal images. Scheme of slices lateral view (a), cross sections of the female (b–j). ab, arcuate body; abd, arcuate body dorsal lobe; abv, arcuate body ventral lobe; cbr, cell body rind; ch, chelicerae; dl, m. dilator proventriculi lateralis; eso, esophagus; fl, m. cheliceral flexor; ln1-ln4, neuropils of the legs 1-4; mb, mushroom body; mbbr, mushroom body bridge; nch, cheliceral neuropil; npp, pedipalpal neuropil; oc, eyes; opin, opisthosomal nerve; ss, sucking stomach; vg, venom gland; vnaml, anterior medial visual neuropil (lamina); vnamm, anterior medial visual neuropil (medulla); vnpl, posterior lateral visual neuropil.

Table S1. Comparison of the prosomal muscles of *Rayforstia* and some other spiders (Palmgren, 1978) : no data (?), muscle present (+), muscle absent (−).

Name of the muscle	Cheliceral muscles										Pedipalpal muscles										Muscles associated with the digestive system										Endosternite, leg muscles																							Muscles originating from the carapace, without any contact with the legs
	al	av	ex	fl	la	lp	mc	me	pd (m. medialis pro-descendens)	rd (m. medialis retro-descendens)	ai	as	pa	pe	pi	pm	pp	ps	ips	ipi	crv	da	dp	da+ dp	dd	dl	dphl	rl	rls	rli	ca	cb	cc	c1 (m. tergo-coxalis anterior profundus)	c2 (m. t-c medius anterior)	c3 (m. t-c medius posterior)	c4 (m. t-c posterior profundus)	c5 (m. endosterno-coxalis antero-superior)	c5+c7	c6 (m. e-c postero-superior)	c7	c7+c8	c8	ed (m. plagulo endosternalis dorsalis)	ed+ev (m. plagulo endosternalis dorsalis and ventralis)	el (not in abbreviations, only in pictures)	ep	ev (m. plagulo endosternalis ventralis)	le	lt (m. loro-tergalis)	pt (m. plagulo-tergalis)	sc	s1-4	ML (m. lateralis)
Rayforstia sp. (896 µm), ♀, Anapidae	+	+	+	+	+	+	+	-	-	+	-	-	+	+	-	+	+	-	+	+	+	-	-	+	+	+	+	+	+	+	+	+	+	-	-	-	-	-	-	-	+	-	+	-	-	-	+	-	+	-	-	+	+	-
Comaroma simoni (1.6 mm), ♀, Anapidae	+	+	+	+	+	+	+	+	+	+	+	+	+	-	+	+	+	+	-	-	?	+	+	-	+	?	?	?	-	-	+	+	+	-	-	-	-	-	-	-	+	-	+	+	-	-	+	+	+	+	+	+	+	+
Parasteatoda lunata (2.7-5 mm), ♂, Theridiidae	+	+	+	+	+	+	+	+	+	+	+	+	+	-	+	+	+	+	-	-	?	-	-	+	+	?	?	?	-	-	-	-	-	+	+	+	-	+	-	+	+	-	+	+	-	+	-	+	-	?	+	+	+	+
Theridion pictum (3.5-4.8 mm), ♀, Theridiidae	+	+	+	+	+	+	+	+	+	+	+	+	+	?	?	+	+	+	-	-	?	+	+	-	+	?	?	?	-	-	-	-	-	+	+	+	+	+	-	+	+	-	+	+	-	-	+	+	+	+	+	+	+	+
Minyriolus pusillus (1.1-1.4 mm), ♀, Linyphiidae	+	+	+	+	+	+	+	+	+	+	?	?	+	?	+	+	+	?	-	-	?	-	-	+	+	+	?	?	-	-	-	-	-	+	+	+	+	+	-	+	-	-	-	+	-	-	+	+	+	+	+	+	+	+
Notioscopus sarcinatu s (1.8 mm), ♂, Linyphiidae	+	+	+	+	+	+	+	+	+	+	?	+	+	?	?	+	+	+	-	-	?	-	-	+	+	+	?	?	-	-	-	-	-	+	+	+	-	+	-	+	+	-	+	+	-	+	+	+	-	+	+	+	+	+
Diplocephalus latifrons (1.5-1.9 mm), ♂, Linyphiidae	+	+	+	+	+	+	+	-	+	+	?	?	+	?	?	+	+	?	-	-	?	-	-	+	+	?	?	?	-	-	-	-	-	+	+	+	?	+	-	+	+	-	+	+	-	+	?	+	-	+	+	+	+	?
Diplocephalus latifrons (1.5-2 mm), ♀, Linyphiidae	+	+	+	+	+	+	+	+	+	+	?	+	+	?	+	+	+	+	-	-	?	+	+	-	+	?	?	?	-	-	-	-	-	+	+	+	+ (ML+c4)	+	-	+	+	-	+	-	+	+	+	-	-	+	+	+	+	+ (ML+c4)
Savignia frontata (1.5-2 mm), ♂, Linyphiidae	+	+	+	+	+	+	+	+	+	+	+	+	+	?	+	+	+	+	-	-	?	+	+	-	+	?	?	?	-	-	-	-	-	+	+	+	-	+	-	+	+	-	+	+	-	+	+	+	-	+	+	+	+	+
Savignia frontata (1.5-2 mm), ♀, Linyphiidae	+	+	+	+	+	+	+	+	+	+	+	+	+	?	+	+	+	+	-	-	?	-	-	+	+	+	?	?	-	-	-	-	-	+	+	+	-	+	-	+	+	-	+	+	-	+	+	+	-	+	+	+	+	+
Dismodicus bifrons (1.7-2.1 mm), ♂, Linyphiidae	+	+	+	+	+	+	+	+	+	+	+	+	+	-	+	+	+	+	-	-	?	-	-	+	+	?	?	?	-	-	-	-	-	+	+	+	-	+	-	+	+	-	+	+	-	-	+	+	-	+	+	+	+	+
Oedothorax retusus (2-2.2 mm), ♂, Linyphiidae	+	+	+	+	+	+	+	+	+	+	+	+	+	?	+	+	+	+	-	-	?	-	-	+	+	?	?	?	-	-	-	-	-	+	+	+	-	+	-	+	+	-	+	+	-	+	+	+	-	+	+	+	+	+
Oedothorax gibbosus (2-2.2 mm), ♂, Linyphiidae	+	+	+	+	+	+	+	+	+	+	+	+	+	?	+	+	+	+	-	-	?	-	-	+	+	+	?	?	-	-	-	-	-	+ (ML+c1)	+	+	-	+	-	+	+	-	+	+	-	-	+	+	+	+	+	+	+	+ (ML+c1)
Centromerus incilium (2.2-2.5 mm), ♀, Linyphiidae	+	+	+	+	+	+	+	+	+	+	+	+	+	?	+	+	+	+	-	-	?	+	-	-	+	?	?	?	-	-	-	-	-	+	+	+	-	+	-	+	+	-	+	+	-	+	+	+	-	+	+	+	+	+
Walckenaeria unicornis (2-2.5 mm), ♀, Linyphiidae	+	+	+	+	+	+	+	+	+	+	?	+	+	?	+	+	+	+	-	-	?	+	+	-	+	?	?	?	-	-	-	-	-	+	+	+	-	+	-	+	+	-	+	+	-	-	+	+	+	+	+	+	+	+
Hypomma bituberculatum (2.2-2.5 mm), ♂, Linyphiidae	+	+	+	+	+	+	+	+	+	+	?	+	+	?	+	+	+	+	-	-	?	-	-	+	+	+	?	?	-	-	-	-	-	+	+	+	+	+	-	+	+	-	+	+	-	+	+	+	-	+	+	+	+	?
Erigone arctica (2.5-3.2 mm), ♂, Linyphiidae	+	+	+	+	+	+	+	+	+	+	+	+	+	?	+	+	+	+	-	-	?	+	+	-	+	?	?	?	-	-	-	-	-	+	+	+	-	+	-	+	+	-	+	+	-	+	+	+	-	+	+	+	+	+
Hypomma bituberculatum (2.5-3 mm), ♀, Linyphiidae	+	+	+	+	+	+	+	+	+	+	?	+	+	?	?	+	+	+	-	-	?	-	-	+	+	+	?	?	-	-	-	-	-	+	+	+	+	+	-	+	+	-	+	+	-	+	?	+	-	+	+	+	+	?
Kaestneria dorsalis (2.5-3 mm), ♂, Linyphiidae	+	+	+	+	+	+	+	+	+	+	+	+	+	-	+	+	+	+	-	-	?	+	-	-	+	?	?	?	-	-	-	-	-	+	+	+	-	+	-	+	+	-	+	-	+	+	?	-	-	+	+	+	+	?
Kaestneria dorsalis (2.5-3 mm), ♀, Linyphiidae	+	+	+	+	+	+	+	+	+	+	+	+	+	-	+	+	+	+	-	-	?	-	-	+	+	?	?	?	-	-	-	-	-	+	+	+	-	+	-	+	+	-	+	+	-	-	+	+	+	+	+	+	+	?
Bolyphantes alticeps (3-4.5 mm), ♂, Linyphiidae	+	+	+	+	+	+	+	+	+	-	+	+	+	?	+	+	+	+	-	-	?	+	-	-	+	?	?	?	-	-	-	-	-	+	+	+	-	+	-	+	?	-	+	-	+	+	?	-	-	-	+	+	+	+
Zornella cultrigera (3.2-3.7 mm), ♂, Linyphiidae	+	+	+	+	+	+	+	?	+	+	+	+	+	-	+	+	+	+	-	-	?	-	-	+	+	?	?	?	-	-	-	-	-	+	+	+	-	+	-	+	+	-	+	+	-	+	-	+		+	+	+	+	+
Macrargus rufus (3-6 mm), ♀, Linyphiidae	+	+	+	+	+	+	+	+	+	+	+	+	+	-	+	+	+	+	-	-	?	+	-	-	+	?	?	?	-	-	-	-	-	+	+	+	-	+	-	+	+	-	+	+	-	+	+	+	-	+	+	+	+	?
Stemonyphantes lineatus (4.5-7.0 mm), ♀, Linyphiidae	+	+	+	+	+	+	+	+	+	+	+	+	+	-	+	+	+	+	-	-	?	+	+	-	+	?	?	?	-	-	-	-	-	+	+	+	-	-	+	+	-	-	+	+	-	+	+	+	-	+	+	+	+	+
Linyphia tenuipalpis (4-7.5 mm), ♀, Linyphiidae	+	+	+	+	+	+	+	+	+	+	?	?	+	?	?	+	+	?	-	-	?	+	-	-	+	?	?	?	-	-	-	-	-	+	+	+	?	-	-	+	-	-	+	+	-	-	+	+	+	+	+	+	+	+
Neriene montana (8-8.5 mm), ♀, Linyphiidae	+	+	+	+	+	+	+	+	+	+	-	+	+	-	+	+	+	+	-	-	?	+	+	-	+	?	?	?	-	-	-	-	-	+	+	+	-	+	-	+	+	-	+	-	+	-	+	-	+	+	+	+	+	?
Ero furcata (2.5-4.8 mm), ♀, Mimetidae	+	+	+	+	+	+	+	+	+	+	+	+	+	-	+	+	+	+	-	-	?	+	+	-	+	?	?	?	-	-	-	-	-	+	+	+	?	+	-	+	+	-	+	-	+	-	+	-	+	+	+	+	+	+
Pachygnatha listeri (4.5-5 mm) ♀, Tetragnathidae	+	+	+	+	?	?	+	+	+	+	+	+	+	?	+	+	+	+	-	-	?	+	+	-	+	+	?	?	-	-	-	-	-	+	+	+	-	+	-	+	-	+	-	+	-	-	+	+	+	+	+	+	+	+
Metellina segmentata (6.5-9 mm) ♀, Tetragnathidae	+	+	+	+	+	+	+	?	+	+	?	?	+	?	?	+	+	?	-	-	?	+	+	-	+	-	?	?	-	-	-	-	-	+	+	+	-	+	-	+	+	-	+	+	-	+	?	+	-	+	+	+	+	?
Tetragnatha extensa (5-12.2 mm) ♀, Tetragnathidae	+	+	+	+	+	+	+	+	+	+	+	+	+	?	+	+	+	+	-	-	?	-	-	+	+	?	?	?	-	-	-	-	-	+	+	+	-	+	-	+	+	-	+	+	-	-	+	+	+	+	+	+	+	+
Tetragnatha extensa (5-12.2 mm) ♂, Tetragnathidae	+	+	+	+	+	+	+	+	+	+	+	+	+	?	+	+	+	+	-	-	?	-	-	+	+	?	?	?	-	-	-	-	-	+	+	+	-	+	-	+	+	-	+	+	-	-	+	+	+	+	+	+	+	+
Larinioides cornutus (6.0-20.0 mm), ♀, Araneidae	+	+	+	+	+	+	+	+	+	+	?	+	+	-	+	+	+	+	-	-	?	+	+	-	+	?	?	?	-	-	-	-	-	+	+	+	+	+	-	?	+	-	?	-	+	-	+	-	+	+	+	+	+	+
