# Supplementary material for: Miniaturization does not change conserved spider anatomy, a case study on spider Rayforstia (Araneae: Anapidae)
Source: Sci Rep. 2023 Oct 11;13:17219. doi: 10.1038/s41598-023-44230-3 (PMC10567922; doi:10.1038/s41598-023-44230-3)

Supplement 2. Anatomy of the spider *Rayforstia*, 3D model.

3D models of organ systems of the spider *Rayforstia* are present. The list of organ systems and structures is available in the element tree.

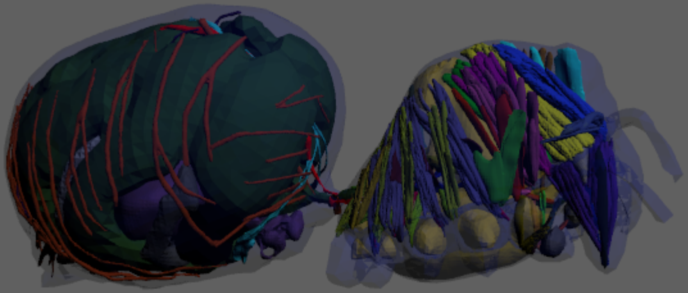

Supplement: Supplementary file 2 — Supplementary Information 2. [file 41598_2023_44230_MOESM2_ESM.pdf]
